# National Health Insurance unpacked: Part 4: Remuneration of practitioners

**DOI:** 10.4102/safp.v62i1.5241

**Published:** 2020-11-04

**Authors:** Robert Mash

**Affiliations:** 1 Division of Family Medicine and Primary Care, Faculty of Medicine and Health Sciences, University of Stellenbosch, Cape Town, South Africa

## Introduction

This four-part series in the *South African Family Practice* journal unpacks the details of National Health Insurance (NHI) as proposed in the NHI Bill that went before Parliament in July 2019.^1^

In the final part, Part 4, of the series, we look at the remuneration of primary care doctors. The NHI Bill gives a very broad overview of the principles involved, but little real detail. The details provided in this article are based on presentations from the Department of Health and National Treasury as well as input from Dr Nicholas Crisp. However, much of these details remain under discussion and need to be piloted to test the feasibility.

## How will remuneration levels be calculated?

**FIGURE 1:**
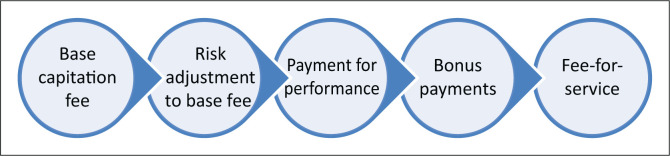
Determination of remuneration.

Figure 1 describes the five components of a system that will influence the remuneration of providers. A key principle will be to keep the system as simple as possible initially and build in more complexity as it evolves. The model is a hybrid with the major component based on risk-adjusted capitation and the minor component based on performance. A hybrid model attempts to balance the pros and cons of different approaches.

Remuneration levels will be based on a capitation model where a base fee is paid per person registered. It seems that this will be calculated at the level of the Contracting Unit for Primary Health Care (CUP) for the population served by a district hospital and will also be adjusted for risk as explained below. In essence, this will be the core funding given to the CUP and made available to the primary care establishments or providers accredited by the CUP in their catchment area.^2^ At present, it seems that CUPs will be given some flexibility to decide on the model by which they then remunerate their accredited establishments or providers. To me, this seems an important area of uncertainty that needs more clarification. There is an implication that if a population is over serviced with too many accredited establishments or providers, then there will be less funding per provider available and vice versa. A number of factors will be compared to determine the base fee. These factors include the current average costs of a patient in the public sector and the current fees-for-service charged in the private sector and research studies.

The base fee will be adjusted for the health risks inherent in the population served. Some populations have more health risks and needs than others, which translates into more visits and higher costs. The age and sex profile of the population will be important in making this adjustment as common conditions such as human immunodeficiency virus (HIV), non-communicable diseases and pregnancy are related to this profile. It may also be necessary to adjust for rurality and, once data allow, for the actual morbidity profile of the population served.

In addition to the adjusted base rate, there will also be an adjustment for performance. The aim is to measure improvements in health outcomes related to performance, for example, admissions to hospital for diabetic foot complications. Measurements will be kept to the minimum and be as practical as possible. It is likely that the adjustment for annual performance will be meaningful in order to incentivise improvement. Measures such as, referral or admission rates and utilisation of investigations or medication will relate to the extent and cost of care by a practitioner. It may also be possible to look at increasing coverage of underserved communities. Measures of patient experience (patient reported outcome measures), control of chronic conditions and proportion of eligible patients covered (e.g. for cervical cancer, immunisations) will relate to the quality of care. Finally, it is possible that selected activities will attract a fee-for-service in order to improve the quality of care in certain areas.

To my mind, providers should also be rewarded for additional relevant qualifications and expertise. Currently, family physicians with postgraduate qualifications and training are registered and paid as specialists. It is not clear how they will be remunerated under NHI, but maybe the concept of a bonus payment should be considered. The national Postgraduate Diploma in Family Medicine also re-orientates and upskills primary care doctors for the future health system and enhances clinical competence and in my view should also be incentivised in remuneration under NHI.

## Who is remunerated – The practice or the practitioner?

The entity that is contracted with the CUP will be remunerated. This could be a health establishment or a primary care provider, but the juristic entity has not yet been decided.

For the individual practitioner, a balance will be struck between the certainty of income from the capitated and risk-adjusted major share of the budget and the incentives provided by the performance-related components of the budget.

## Who will be employing doctors in the future and who will be paying them?

At present, it appears that providers will continue to be employed in the public or private sectors. In the public sector, the government will employ providers, and they would receive a salary which was dependent on funds received from the CUP and performance. In the private sector, providers may be employed through a variety of mechanisms, for example, by a private health service or as a partner in a general practice, and this is not envisaged to change.

## What happens if you don’t get paid timeously?

Payment by the CUP to health establishments or providers would be regular on a weekly or monthly basis. Until the juristic entity is clearly defined, it is not yet known who will hold the bank account and effect individual payments.

## How will medication be procured and dispensed in these practices?

The Office of Health Products Procurement (OHPP) will be established to procure medicines, medical devices and equipment at a national level. The exact local mechanism is still being debated, but one viewpoint is that the money for medication will be included in the funds available to the CUP. They will then purchase medication and supplies from the OHPP approved suppliers at set prices. Establishments or providers will report on purchases claimed and volumes used as part of the information system requirements to ensure that there is no fraud.

## How will practices access laboratory and imaging services?

National Health Laboratory Services designated for primary care and paid for by the fund will be available to all accredited primary care establishments and providers. It is possible that private laboratory services may also be accredited.

Payment systems for imaging services are not yet clear. Currently, some large primary care facilities have their own radiography services, while others would need to refer to a hospital or radiology service for this.
